# Development and Evaluation of a Monocular Camera–Based Mobile Exergame for at-Home Intervention in Individuals at High Risk of Type 2 Diabetes: Randomized Controlled Trial

**DOI:** 10.2196/75823

**Published:** 2025-12-12

**Authors:** Jianan Zhao, Dian Zhu, Yanan Wang, Yaqin Xia, Zeshi Zhu, Jihong Yu

**Affiliations:** 1 College of Fashion and Design, Donghua University, East Yanan Road 1882, Shanghai, 200240, China, 86 18200484800; 2 School of Design, Shanghai Jiao Tong University, Shanghai, China

**Keywords:** monocular camera, exergames, type 2 diabetes, gamification, disease prevention

## Abstract

**Background:**

Exergames have emerged as effective interventions for promoting physical activity and preventing type 2 diabetes (T2D). Kinect-based exergames have demonstrated improvements in exercise adherence and health outcomes, but their high cost and reliance on specialized hardware hinder widespread home-based adoption. Recent advances in computer vision now enable monocular camera–based systems, offering a potentially cost-effective and scalable alternative for promoting physical activity at home.

**Objective:**

This study aimed to evaluate the feasibility and user experience of monocular camera–based exergames as a home-based intervention for individuals at risk for T2D.

**Methods:**

Forty-five community-dwelling individuals at high risk for T2D (mean age 47.12, SD 6.92 years) were recruited and randomized into three groups (n=15 each): (1) control group (traditional offline exercise), (2) Kinect group (Kinect-based exergame), and (3) monocular group (monocular camera–based exergame). Participants engaged in a 10-minute intervention once per week for 7 weeks. Data were collected at 3 time points: baseline (exercise performance: heart rate and perceived fatigue), postintervention (exercise performance and user experience, including game experience and intrinsic motivation), and follow-up (user engagement and qualitative feedback). One-way ANOVA was used for data analysis.

**Results:**

Exercise performance was comparable across all groups, with no significant differences in heart rate (*P*=.76) or fatigue levels (*P*=.25). However, participants in the monocular group reported significantly lower fatigue than those in the control group (*P*=.04). Intrinsic motivation was significantly higher in both the Kinect (mean 35.13, SD 3.20) and monocular (mean 34.00, SD 4.41) groups than in the control group (mean 26.06, SD 1.87; *P*<.001), with no significant difference between the 2 exergame groups (*P*=.44). While most user experience measures showed no significant differences, the monocular group reported a higher perceived challenge (mean 3.45, SD 0.51) than the Kinect group (mean 2.96, SD 0.39; *P*=.09). Additionally, the monocular group exhibited higher engagement, as evidenced by more frequent use, fewer challenges, and a greater intention to continue using the system.

**Conclusions:**

Monocular camera–based exergame is a feasible and effective solution for promoting physical activity in individuals at risk for T2D. It offers motivational and experiential benefits similar to Kinect-based systems but requires less costly and more accessible equipment. These findings suggest that monocular systems have strong potential as scalable tools for home-based chronic disease prevention.

## Introduction

Type 2 diabetes (T2D) is a chronic, progressive condition that poses a major global health challenge, with the number of affected individuals projected to reach 500 million by 2030 [1,2]. The mean age of T2D diagnosis is 45 years [3]. Individuals older than 35 years, those with obesity, having a family history of diabetes, not physically active, and having prediabetes are considered to have high risk of T2D [4]. Among high-risk populations, lifestyle interventions, particularly those that encourage physical activity, are crucial for delaying or preventing T2D onset [5]. Despite the proven benefits of exercise, barriers such as lack of motivation, limited access to fitness resources, and insufficient guidance persist [6,7]. To address these challenges, technology-driven solutions such as digital exergames have emerged as promising tools to promote physical activity in an engaging and accessible way [8].

Exergames combine exercise with interactive gaming and offer real-time feedback, personalized experiences, and gamified rewards, all of which enhance physical activity [9]. Motion-sensing technology has been central to the development of exergames, with Microsoft’s Kinect being a landmark innovation [10]. Released in 2010, Kinect revolutionized motion-sensing games by enabling real-time body tracking and virtual avatar control through natural movements [11]. Kinect-based exergames have been extensively studied for their impact on physical performance, therapy adherence, and clinical outcomes [12]. However, due to reliance on specialized hardware, high costs, ineffective marketing strategies, and limited home usability, the Kinect v2 was discontinued in 2023.

In recent years, advancements in deep learning have enabled a new generation of motion-sensing technologies, including monocular human pose tracking, which relies solely on monocular cameras [13]. This approach allows for real-time mapping of users’ movements in virtual environments without requiring expensive depth cameras or wearable devices [14].

The monocular camera–based exergame emerges as a low-cost, noncontact technology and offers a practical solution for home-based exergame training [15]. Its potential for enhancing accessibility and usability makes it an attractive area for exploration. However, while the potential of monocular camera–based pose tracking has been identified, its use in designing and evaluating exergames tailored to patients with T2D remains rarely explored, and the quality assessment of the exergame is rarely discussed as well [16,17]. This creates a need for accessible, scalable solutions that not only use cutting-edge technologies but also align with the practical needs and preferences of the target audience.

To address these gaps, this study introduces an exergame specifically designed for individuals at high risk of diabetes, using monocular camera technology to ensure affordability and ease of use. The game is informed by evidence-based physical activities to promote metabolic health and incorporates features to enhance user engagement and long-term adherence. This study aims to evaluate the effectiveness and user experience of monocular camera–based exergame to understand its potential to replace Kinect in providing home-based exercise intervention. The primary outcome of this study is to demonstrate that there is no significant difference in physical activity levels between the experimental and control groups, as evidenced by the lack of variation in heart rate changes before and after the intervention. The secondary outcome focuses on evaluating the impact of the monocular camera–based group on user experience, providing insights into the acceptability and usability of the intervention from the participants’ perspective.

## Methods

### Study Design

This 3-arm randomized controlled trial used a 1:1:1 allocation ratio to evaluate the effectiveness of an exergame designed using monocular camera technology for individuals at high risk of developing T2D. A total of 45 participants meeting specific inclusion criteria were recruited and randomly assigned to 1 of 3 groups: a control group performing traditional video-guided exercises, a Kinect-based exergame group, and a monocular camera–based exergame group. The sample size was calculated based on a power analysis with an expected medium size at 0.48, where previous research reported medium effect sizes (0.40‐0.50) in evaluating usability and adherence in Kinect-based exergames for individuals with prediabetes [18], aiming for 80% power and a significance level of .05 to detect meaningful differences between the groups. The intervention was conducted in a controlled setting, ensuring consistency across groups while collecting physiological and experiential data.

### Recruitment

A total of 45 individuals at high risk for T2D were recruited through offline outreach facilitated by community managers affiliated with local residential service centers in Minhang District, Shanghai, China. They supported recruitment by identifying eligible participants through existing health records and outreach channels. The inclusion criteria were as follows: (1) 35 years of age and older; (2) a score of 25 or higher on the Chinese Diabetes Risk Score; (3) community-dwelling individuals, not residing in assisted living or long-term care facilities; (4) physically capable of engaging in light to moderate exercise, as determined by self-report and physician clearance; (5) normal cognitive function that enables the participant to complete the experiment independently or with minimal assistance; and (6) written informed consent provided by participants or their families. Exclusion criteria included individuals with (1) diagnosis of T1D or T2D, (2) current participation in another exercise intervention study, (3) severe cognitive impairment with Mini-Mental State Examination score of <24, and (4) major mobility limitations such as severe osteoarthritis and recent orthopedic surgery.

### Experimental Setting and Equipment

#### Experiment Group (Monocular Camera–Based Exergame Design)

##### Overview

This intervention is an exercise game tailored for individuals at high risk of diabetes. It incorporates 6 movements adapted from the “Diabetes Health Exercises,” which include toe stretches, high knees, side arm raises, punching exercises, arm stretches with hip extensions, and elbow-to-chest expansions. These movements were selected for their simplicity, defined training objectives, and distinct characteristics that facilitate recognition and replication. The exercises aim to enhance peripheral blood circulation and target key muscle groups such as the triceps, biceps, gluteal muscles, and lower limbs. Each session is structured to last 10 minutes, comprising 5 minutes of aerobic exercise and 5 minutes of resistance training, thereby providing a balanced regimen that aligns with the physiological needs of the target population.

**Figure 1.**
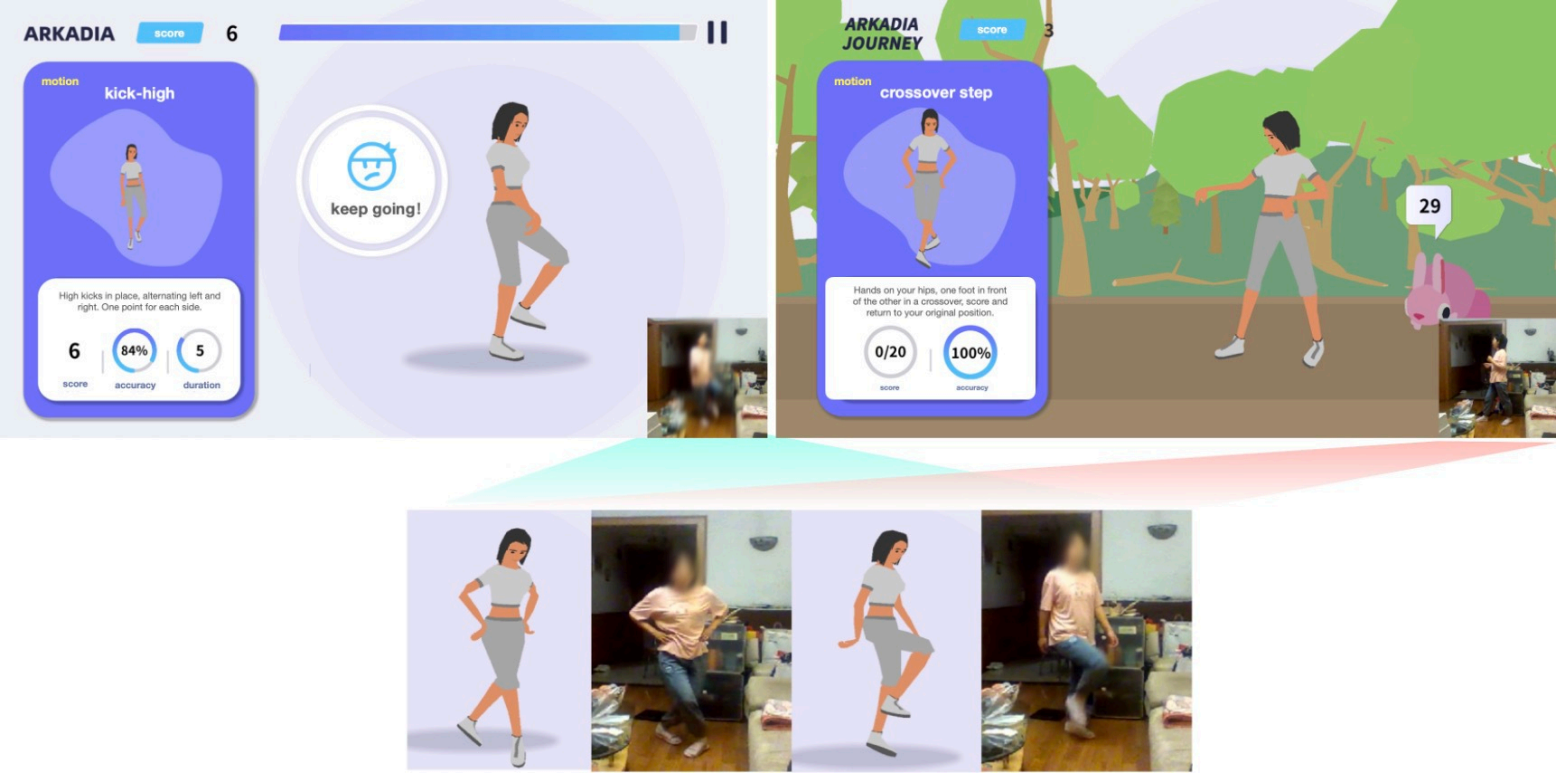
Interface design of the exergame. The exergame interface includes real-time avatar mirroring, performance feedback indicators, instructional visuals, and a reward progress tracker.

The game uses a virtual avatar to replicate users’ movements, thereby fostering an immersive and engaging experience (Figure 1). A monocular camera captures users’ movements in real time, which are analyzed through pose estimation algorithms and subsequently mapped onto the virtual avatar. Users interact with the game by following on-screen visual demonstrations, presented as either static images or animations, to perform the prescribed exercises. Movement accuracy is evaluated by the system, with scores awarded based on performance. To enhance user motivation and adherence, the game incorporates a reward system, where points earned through accurate execution can be redeemed for in-game rewards, such as unlocking background music, avatar customization options, and new virtual environments.

##### Technical Framework of the Monocular Camera–Based Exergame

The intervention is designed with a robust technical foundation, using Figma for interface design and Unity 2019.4 for game development. The motion capture system leverages the open-source ThreeDPoseTracker framework, based on VNect technology, which processes low-resolution red, green, and blue video streams using Unity’s Barracuda system and a pretrained ONNX model. This framework accurately detects 10 critical body landmarks—wrists, elbows, ankles, knees, waist, and head—estimating their 3D spatial positions in real time.

The real-time pose correction process compares the user’s detected body posture with a predefined regulated posture within the exergame. The correction formula involves angular calculations between vectors formed by detected body landmarks, as shown in the image. Specifically, each detected angle (θ_i_) is compared with the corresponding regulated angle (θ′_i_), and the overall difference (*D*_2_) is calculated as *D*_2_=∑(θi−θ′_i_). To quantify the degree of correction required, a recognition threshold parameter (*D*_st_) and a baseline recognition rate parameter (*S*_st_) are used. The final evaluation metric (*S*) is determined by the piecewise function:


$$S=\left\{\begin{array}{cc} D_{st}-D\;\times \frac{100-S_{st}}{D_{st}}+S_{st}, & 0\leq D<D_{st}\\ 0, & D>D_{st} \end{array}\right\}$$

This ensures that minor deviations from the regulated posture are dynamically adjusted, while significant deviations are flagged as errors. Temporal filtering techniques are applied to improve the smoothness and precision of pose estimations, which are then mapped to a skeletal model in Unity for seamless motion replication.

By integrating real-time motion capture and precise pose correction, the system provides immediate feedback, ensuring accurate and adaptive user movements during gameplay. This enhances the usability and efficacy of the intervention, making it a scientifically grounded and technologically advanced solution for promoting physical activity, particularly for diabetes prevention.

### Kinect Group

Kinect group features the same software functionality and design as the application based on bullet-screen cameras, but the hardware is built using a Kinect sensor. The gamified platform is primarily developed and presented using Unity 3D. The Kinect 3D motion-sensing camera incorporates real-time dynamic capture and image recognition capabilities, offering new possibilities for interactive approaches to motion therapy.

### Control Group

The control group followed a set of 6 instructor-led exercise videos, which included crossover steps, high knees, lateral raises, punching movements, downward leg punches from a standing position, and elbow-to-chest expansions. Each session in the control group was designed to reflect the same aerobic and resistance exercise level as the Kinect group and the experimental group, ensuring comparability across groups in terms of physical exertion and activity content.

### Procedure

Participant recruitment was conducted from March to April 2024 in the Hongqiao Community. Individuals meeting the inclusion criteria were provided with comprehensive information about the study and signed written informed consent. Baseline demographic data were collected prior to random assignment using block randomization by researcher into one of three groups: (1) control group (traditional exercise control), (2) Kinect group (Kinect-based exergame), or (3) monocular group (monocular camera–based exergame).

Three days prior to the intervention, all participants received instructional videos demonstrating the 6 targeted movements to be performed during the exercise sessions. Participants were instructed to review and practice these movements to ensure familiarity with the protocol.

On the day prior to the intervention, trained researchers and caregivers delivered and assisted in setting up the required equipment—either a Kinect V2 sensor or an Aoni C33 monocular camera (manufactured by Shenzhen Aoni Electronic Industry Co, Ltd, resolution: 1920×1080 pixels, frame rate: 30 fps, field of view: 90°)—in participants’ homes. Setup was scheduled in the afternoon, approximately 1‐2 hours post meal, to ensure participants’ comfort and safety during physical activity.

On the first intervention day, baseline data, including age, gender, T2D risk, resting heart rate, and perceived fatigue, were measured prior to the session. Each intervention session lasted 10 minutes and consisted of 2 components: 5 minutes of aerobic exercise followed by 5 minutes of resistance training. Participants in the control group followed a prerecorded instructional video demonstrating the 6 aerobic movements, followed by guided resistance exercises. The monocular group used a custom-developed exergame based on monocular camera technology. Real-time visual instructions were displayed on a television screen, guiding participants through the same movement protocol. The Kinect group used an exergame designed with Kinect-based skeletal tracking, offering a similar visual and instructional format as the monocular system.

During the aerobic component, participants performed 6 distinct movements, each lasting 50 seconds. For the resistance component, participants selected their preferred resistance level (ranging from 0.25 to 1.5 kg), and resistance bands were applied to the arms and legs under caregiver supervision. Caregivers and researchers refrained from intervening during the session unless directly requested by participants to minimize external influence.

Immediately following the first intervention, participants’ postexercise heart rate, perceived fatigue (via Borg Rating of Perceived Exertion Scale), intrinsic motivation (via Intrinsic Motivation Inventory [IMI]), and user experience (including perceived enjoyment, challenge, and usability) were assessed using standardized instruments.

Participants were then instructed to continue the 10-minute intervention daily for 7 consecutive days independently without additional notification from the researchers. They were asked to document their participation by recording short videos as proof of session completion. At the end of the 1-week period, researchers returned to collect the devices and retrieve adherence data, including the number of completed sessions and any user feedback.

### Outcome Measure

#### Exercise Performance

To evaluate the amount of exercise during the experiment, heart rate and perceived fatigue were assessed among participants at the baseline and immediately after the intervention. The postintervention heart rate was collected during the first 10 seconds postexercise to estimate physiological responses. Considering the general physical condition of individuals at high risk of diabetes, the study used 50%‐80% of the maximum heart rate as the target exercise intensity. The maximum heart rate was calculated using the formula: 208 − (Age × 0.7). Additionally, perceived fatigue, a widely accepted parameter in exercise assessments for diabetes-related fields, was used to supplement the evaluation of physical activity. The Borg Rating of Perceived Exertion Scale [19], ranging from 6 to 20, was used to assess subjective fatigue and compare perceived exercise intensity across groups.

#### Perceived User Experience

At the first session of intervention, participants in the Kinect and monocular groups completed the Game Experience Questionnaire (GEQ) to assess their user experience [20]. The GEQ was used to evaluate and compare the impact of different technologies on immersion and the overall experience of exergames. Furthermore, the Interest/Enjoyment Subscale of the IMI was administered to participants in the control group, Kinect group, and monocular group [21]. This subscale evaluated and compared intrinsic motivation and enjoyment associated with physical activity across the 3 groups. At the end of the 1-week experiment period, user engagement was further quantified by tracking the frequency of intervention use over the 1-week period, based on participants’ video-recorded usage logs. To complement the quantitative data, a brief qualitative assessment was conducted at the end of the intervention week. Participants responded to open-ended questions such as: “Did you experience any issues while using the exergame?” and “Would you be interested in continuing to use the exergame in the future?”

### Data Analysis

Data collected from the physiological measurements (resting and exercise heart rates) and the questionnaires were analyzed to compare the differences between groups and evaluate the effectiveness of the interventions. ANOVA analysis was used to understand the differences of the data collected from 3 experiment groups [22], and 2-tailed *t* test (for normally distributed result) or nonparametric test (for non–normally distributed result) was used to understand the significance of differences between 2 groups. For pairwise comparisons between 2 groups, independent-samples 2-tailed *t* tests were applied for normally distributed data. For data not meeting normality assumptions, nonparametric tests were used: the Kruskal-Wallis test for comparisons among 3 groups and the Mann-Whitney *U* test for pairwise group comparisons. Statistical analyses were conducted using SPSS (version 26.0; IBM Corp), with a significance level set at *P*<.05. Cohen *f* value is used to understand the effect size, and the critical points for small, medium, and large effect sizes are 0.1, 0.40, and 0.80, respectively.

### Ethical Considerations

This study was approved by the Shanghai Jiao Tong University’s institutional review board (H20230160I), and all procedures were conducted in accordance with the ethical standards outlined in the Declaration of Helsinki. Due to the initial pilot-phase nature of the project, the trial was retrospectively registered at ClinicalTrials.gov (NCT06950528), prior to data analysis. All participants provided written informed consent prior to participation. The study ensured strict confidentiality of participant data, and no personally identifiable information was collected or disclosed. Participants received a US $7 shopping voucher after completing the study. No identifiable images or recordings were used in the manuscript or supplementary materials.

## Results

### Overview

**Figure 2.**
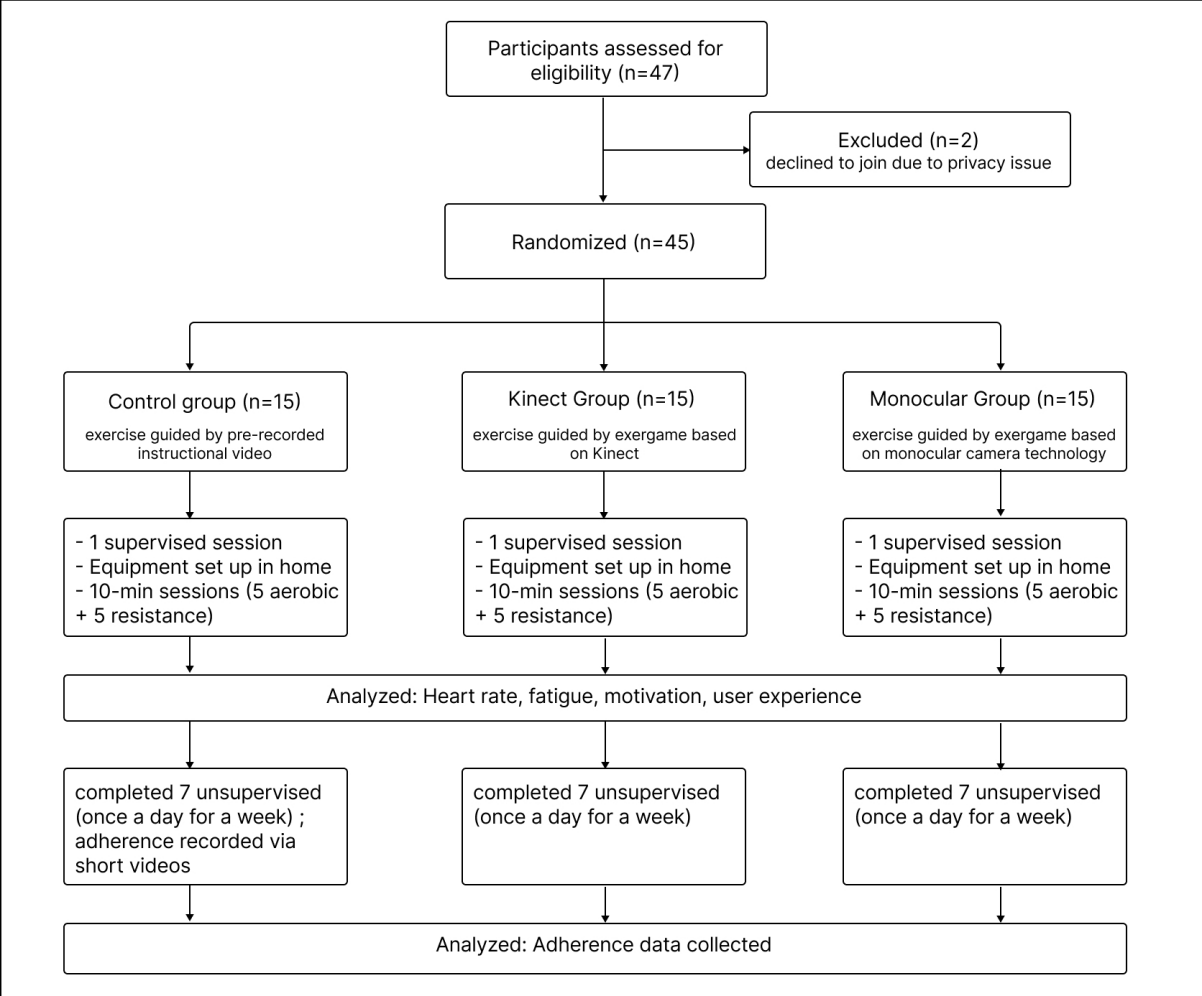
The CONSORT (Consolidated Standards of Reporting Trials) flowchart of the experiment.

This study recruited 45 participants identified as being at high risk for T2D. The CONSORT (Consolidated Standards of Reporting Trials) flowchart is shown in Figure 2. Participants aged between 40 and 54 years, with a mean age of 47.12 (SD 6.92) years. Participants were classified as high-risk based on the Chinese Diabetes Risk Score, with an average mean score of 33.38 (SD 5.04). As shown in Table 1, an ANOVA analysis of baseline characteristics among the 3 participant groups revealed no statistically significant differences. No negative incidents were reported.

**Table 1.** Baseline data of the recruited participants.

Characteristics	Control group (n=15)	Kinect group (n=15)	Monocular group (n=15)	*P* value
Gender (female), n (%)	8 (53.33%)	9 (60.0%)	8 (53.33%)	—^a^
Age (year), mean (SD)	47.27 (6.59)	47.93 (6.85)	46.17 (7.33)	.80
T2D^b^ risk, mean (SD)	33.07 (5.02)	33.60 (4.75)	33.47 (5.36)	.84
Heart rate, mean (SD)	79.46 (10.23)	80.06 (10.01)	78.02 (8.66)	.48
Perceived fatigue, mean (SD)	9.27 (1.53)	9.41 (1.12)	9.28 (1.06)	.81

a Not available.

b T2D: type 2 diabetes.

### Outcomes in Exercise Performance

The amount of exercise was assessed through heart rate and perceived fatigue. As shown in Table 2, the average heart rate for the control group, Kinect group, and monocular group was 86.34 (SD 7.48), 90.81 (SD 7.71), and 89.29 (SD 6.52), respectively. The between-group comparison revealed no significant difference in heart rate (*P*=.76; Cohen *f*=0.117), indicating a small effect size.

Exercise performance was evaluated using 2 indicators: heart rate as an objective physiological measure and perceived fatigue as a subjective self-report metric. As shown in Table 2, the average heart rates for the control, Kinect, and monocular groups were 86.34 (SD 7.48) bpm, 90.81 (SD 7.71) bpm, and 89.29 (SD 6.52) bpm, respectively. A one-way ANOVA revealed no statistically significant differences among the groups (*F*_2,42_=0.28; *P*=.76), with a small effect size (Cohen *f*=0.117). These results suggest that the type of system used did not substantially impact cardiovascular exertion during the exercise session.

**Figure 3.**
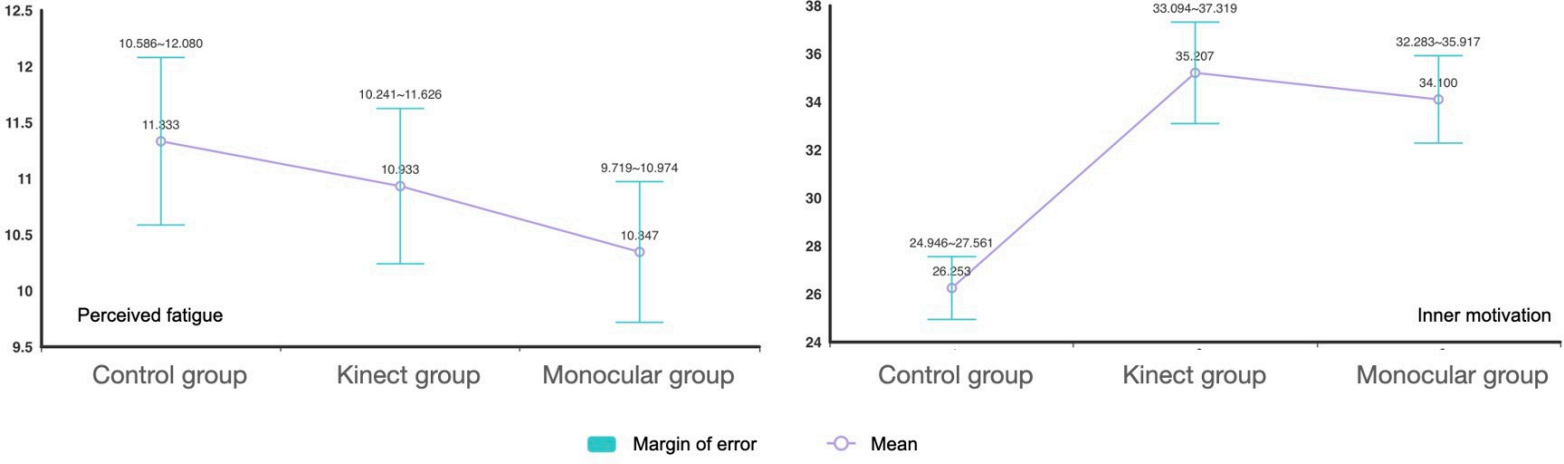
Comparison of perceived fatigue and intrinsic motivation across 3 participant groups.

In terms of perceived fatigue, all participants reported increased fatigue after the intervention (*P*<.05). As shown in Figure 3, the control group reported the highest average fatigue level (mean 11.60, SD 1.12). However, between-group comparisons did not reach statistical significance (*P*=.25; Cohen *f*=0.265), indicating a moderate effect size. Post hoc comparisons revealed that the monocular group experienced significantly lower fatigue than the control group (*P*=.04), while the Kinect group’s difference was not significant (*P*=.15). These findings suggest a potential advantage of the monocular system in reducing perceived exertion during gameplay.

**Table 2.** Data collected before and after the intervention in 3 groups of participants.

Variable	Control group	Kinect group	Monocular group	*F* test (*df*)	*P* value	Cohen *f*
Heart rate				0.28 (2,42)	.76	0.117
Data collected at baseline, mean (SD)	79.46 (10.23)	80.06 (10.01)	78.02 (8.66)			
Data collected after experiment, mean (SD)	86.34 (7.48)	90.81 (7.71)	89.29 (6.52)			
*P* value	.038	.004	.003			
Perceived fatigue				1.442 (2,42)	.25	0.265
Data collected at baseline, mean (SD)	9.27 (1.53)	9.41 (1.12)	9.28 (1.06)			
Data collected after experiment, mean (SD)	11.60 (1.12)	10.60 (1.45)	10.33 (1.49)			
*P* value	<.001	.007	.026			
Inner motivation, mean (SD)	26.06 (1.87)	35.13 (3.20)	34.00 (4.41)	29.701 (2,42)	<.001	1.204

### Outcomes in User Experience

User experience following the first intervention session was assessed through self-reported measures of intrinsic motivation and game experience. Intrinsic motivation scores, as measured by the Interest/Enjoyment subscale of the IMI, were as follows: control group (mean 26.06, SD 1.87), Kinect group (mean 35.13, SD 3.20), and monocular group (mean 34.00, SD 4.41). A one-way ANOVA revealed a significant between-group difference in intrinsic motivation (*P*<.001), with a large effect size (Cohen *f*=1.204). Post hoc comparisons indicated that both the Kinect-based and monocular camera–based exergames significantly enhanced intrinsic motivation compared with those of the control group. However, there was no statistically significant difference between the Kinect and monocular groups (*P*=.44), suggesting comparable motivational effects across the 2 exergaming modalities.

Game experience, as measured by the GEQ and shown in Table 3, revealed significant differences in perceived challenge. Participants in the monocular group reported a higher challenge score (mean 3.45, SD 0.39) than those in the Kinect group (mean 2.96, SD 0.51), with the difference reaching statistical significance (*t*_28_=−2.71; *P*=.01). One possible explanation is the Kinect system’s superior motion-tracking capabilities, which may have facilitated a stronger sense of virtual body ownership and smoother gameplay, thus reducing the perceived difficulty.

**Table 3.** Results of the Game Experience Questionnaire for the Kinect and monocular groups.

Dimension	User feedbacks, mean (SD)		*t* test (*df*)	*P* value
	Kinect group (n=15)	Monocular group (n=15)		
Sensory and imaginative immersion	3.40 (0.38)	3.51 (0.79)	−2.034 (28)	.15
Flow	3.40 (0.54)	3.58 (0.63)	−0.811 (28)	.43
Competence	2.90 (0.60)	3.16 (0.71)	−1.049 (28)	.30
Tension or annoyance	0.10 (0.38)	0.08 (0.28)	0.124 (28)	.90
Challenge	2.96 (0.51)	3.45 (0.39)	−2.71 (28)	.01
Positive affect	3.53 (0.44)	3.79 (0.39)	−1.579 (28)	.13
Negative affect	0.33 (0.67)	0.41 (0.70)	−0.688 (28)	.50

For all other dimensions of game experience—including sensory and imaginative immersion, flow, perceived competence, tension or annoyance, and emotional responses (positive and negative)—no statistically significant differences were observed between the 2 exergame groups (*P*>.05). While not statistically significant, participants in the Kinect group reported slightly higher tension levels, potentially due to unfamiliarity with the hardware. Conversely, the monocular group reported marginally higher negative affect, which may reflect minor limitations in motion-tracking responsiveness or accuracy.

User engagement over the 1-week intervention period was measured by the number of completed exercise sessions. On average, participants in the control group completed 2.33 sessions (SD 1.29), the Kinect group completed 2.93 sessions (SD 1.79), and the monocular group completed 3.60 sessions (SD 1.59). Although the monocular group exhibited the highest level of usage, between-group comparisons did not reach statistical significance (*P*=.49). Similarly, no significant difference was observed between the Kinect and monocular groups (*P*=.29).

A thematic analysis of the qualitative user feedback was conducted to contextualize participants’ experiences across groups. Feedback was first coded and then categorized into three major themes: usability, motivation, and future intention to use. (1) *Usability*: Most participants across all groups found the systems easy to operate. However, specific issues were reported. Four participants in the Kinect group mentioned challenges related to hardware setup (eg, spatial calibration and sensor alignment), while 1 participant in the monocular group cited difficulties with interface responsiveness. (2) *Motivation*: Lack of engagement was a recurring theme in the control group, with 6 participants indicating low motivation due to the static and repetitive nature of the game. (3) *Future intention to use*: Despite these challenges, participants in the exergame groups showed strong willingness to continue. In the Kinect group, 66.67% (10/15) expressed interest in future use, while 80% (12/15) of the monocular group reported a clear intention to continue engaging with the exergames.

## Discussion

### Principal Results

In this study, we evaluated the impact of exercise interventions using 3 modalities—traditional offline exergames, Kinect-based exergames, and monocular camera–based exergames—on exercise performance and user experience in a high-risk T2D population. The findings revealed several noteworthy insights into the potential of monocular camera–based exergames as an affordable and convenient alternative to Kinect-based systems in developing exergame for high-risk T2D population.

From an exercise performance perspective, all 3 modalities—traditional, Kinect-based, and monocular camera–based exergames—elicited comparable elevations in participants’ heart rates, indicating similar levels of cardiovascular engagement. This finding aligns with prior research demonstrating that exergames can effectively promote physical activity across diverse technological platforms [23]. While the heart rate differences did not reach statistical significance, the effect size suggests a small practical effect that may still be meaningful in public health applications, especially for preventive care in at-risk populations.

Notably, participants reported lower perceived fatigue when engaging with digital exergames, particularly the monocular camera–based system, and although these differences did not reach statistical significance, the moderate effect size and significant pairwise difference between the monocular and control groups suggest a clinically relevant trend. This trend suggests that digital interventions may offer a more comfortable exercise experience while maintaining effectiveness, corroborating previous studies that have highlighted the potential of exergames to enhance exercise enjoyment and adherence [24]. The observed lower perceived fatigue in the monocular camera–based exergame group may be attributed to the system’s ability to provide real-time feedback and adjust to users’ movements, thereby enhancing engagement and reducing the subjective experience of exertion. Such features are consistent with findings from studies emphasizing the importance of interactive and adaptive elements in exergame design to optimize user experience and outcomes [25].

### User Experience and Engagement

In terms of user experience, digital exergames were found to foster higher levels of inner motivation than traditional offline exergames, highlighting their potential to engage users more effectively [26,27]. The difference in inner motivation showed a large effect size, indicating the robust motivational potential of digital exercise systems for behavior change. Although there was no significant difference in inner motivation between Kinect-based and monocular camera–based exergames, these 2 systems exhibited distinct strengths in the user experience. Monocular camera–based exergames excelled in providing sensory and imaginative immersion, flow, competence, and positive affect, offering an engaging and enjoyable exercise environment [28]. In contrast, Kinect-based exergames performed better in the dimensions of tension, negative affect, and challenge, with the challenge dimension showing a statistically significant advantage, suggesting Kinect’s ability to reduce negative emotional responses during gameplay [29]. The higher challenge scores in the monocular group may reflect user frustration stemming from occasional inaccuracies in motion tracking or delayed feedback, which can reduce the sense of control and increase cognitive effort.

To enhance the long-term appeal and sustain user engagement, current research indicating incorporating adaptive difficulty levels, personalization features, and reinforcement mechanisms is crucial [30]. Adaptive difficulty can dynamically adjust the game’s challenge to match the user’s skill level, preventing boredom and frustration. Reinforcement mechanisms, including real-time feedback and rewards, can further encourage consistent participation [31]. Future design may enhance these design elements to improve adherence and motivation in exergame interventions.

Nevertheless, participants using the monocular camera system reported fewer setup difficulties and a higher willingness to continue using the system postintervention. Compared with Kinect, the monocular system’s low hardware burden, environmental adaptability, and plug-and-play configuration make it highly promising for home-based and scalable interventions. These findings highlight the practical potential of monocular camera–based exergames as an accessible solution for promoting physical activity in resource-limited settings, where traditional fitness infrastructure or expensive hardware such as Kinect may not be feasible.

### Limitations

Despite encouraging results, this study has several limitations. First, the small sample size and short follow-up duration limit generalizability. Future studies should include larger, more demographically diverse populations. Second, the controlled indoor setup does not fully reflect real-world conditions; testing in-home or community settings is needed to assess ecological validity. Third, the study did not examine long-term adherence or health outcomes (eg, HbA_1c_, weight, and insulin sensitivity), which are critical for evaluating the sustained impact of digital interventions. Fourth, although Kinect-based exergames scored higher in the challenge dimension, participant feedback suggested that the monocular system’s challenge scores may have resulted from tracking errors or interface latency, rather than intentional game difficulty—indicating a need to distinguish between technical and design-related difficulty. Fifth, the study did not conduct subgroup analyses based on gender, which could influence user experience and outcomes due to differences in physical ability, digital familiarity, or motivational preferences. Sixth, the study was originally planned to be conducted at a different location; due to logistical challenges, it was relocated to another community site, which may affect comparability with the original protocol.

Future research should evaluate long-term health outcomes such as HbA_1c_, insulin sensitivity, and weight control associated with these interventions; conduct trials in real-world environments such as community centers or homes to assess ecological validity; investigate personalized game adaptation strategies that adjust challenge and feedback based on individual skill level and fatigue patterns; and develop cross-platform, low-barrier versions of monocular camera–based systems for scalable deployment in resource-limited settings.

### Conclusions

The findings suggest that monocular camera–based exergame offers a viable alternative to Kinect-based exergame for at-home use by T2D high-risk populations. It provides similar exercise outcomes and user experiences while offering the advantages of simplicity and greater adaptability. However, further improvements are needed to enhance user experience, particularly in balancing the challenge dimension.

## Supplementary material

10.2196/75823 Checklist 1 CONSORT-eHEALTH checklist (V 1.6.1).

## Abbreviations

CONSORT: Consolidated Standards of Reporting Trials
CONSORT-EHEALTH: Consolidated Standards of Reporting Trials of Electronic and Mobile Health Applications and Online Telehealth
GEQ: Game Experience Questionnaire
IMI: Intrinsic Motivation Inventory
T2D: type 2 diabetes

## References

[1] Ahmad E, Lim S, Lamptey R, Webb DR, Davies MJ (2022). Type 2 diabetes. Lancet.

[2] Basu S, Yudkin JS, Kehlenbrink S (2019). Estimation of global insulin use for type 2 diabetes, 2018–30: a microsimulation analysis. Lancet Diabetes Endocrinol.

[3] Carrillo-Larco RM, Guzman-Vilca WC, Xu X, Bernabe-Ortiz A (2024). Mean age and body mass index at type 2 diabetes diagnosis: pooled analysis of 56 health surveys across income groups and world regions. Diabet Med.

[4] (2022). Risk factors for type 2 diabetes. National Institutes of Health.

[5] Magkos F, Hjorth MF, Astrup A (2020). Diet and exercise in the prevention and treatment of type 2 diabetes mellitus. Nat Rev Endocrinol.

[6] Dnes N, Coley B, Frisby K (2021). “A little bit of a guidance and a little bit of group support”: a qualitative study of preferences, barriers, and facilitators to participating in community-based exercise opportunities among adults living with chronic pain. Disabil Rehabil.

[7] Farholm A, Sørensen M (2016). Motivation for physical activity and exercise in severe mental illness: a systematic review of intervention studies. Int J Ment Health Nurs.

[8] Choi SD, Guo L, Kang D, Xiong S (2017). Exergame technology and interactive interventions for elderly fall prevention: a systematic literature review. Appl Ergon.

[9] Schättin A, Pickles J, Flagmeier D (2022). Development of a novel home-based exergame with on-body feedback: usability study. JMIR Serious Games.

[10] Huang K, Zhao Y, He R (2022). Exergame-based exercise training for depressive symptoms in adults: a systematic review and meta-analysis. Psychol Sport Exerc.

[11] Hu MC, Chen CW, Cheng WH, Chang CH, Lai JH, Wu JL (2015). Real-time human movement retrieval and assessment with Kinect sensor. IEEE Trans Cybern.

[12] Da Gama A, Fallavollita P, Teichrieb V, Navab N (2015). Motor rehabilitation using Kinect: a systematic review. Games Health J.

[13] Fan Z, Zhu Y, He Y, Sun Q, Liu H, He J (2023). Deep learning on monocular object pose detection and tracking: a comprehensive overview. ACM Comput Surv.

[14] Delibasoglu I, Kosesoy I, Kotan M, Selamet F (2022). Motion detection in moving camera videos using background modeling and FlowNet. J Vis Commun Image Representation.

[15] Chung YY, Annaswamy TM, Prabhakaran B Performance and user experience studies of HILLES: home-based immersive lower limb exergame system.

[16] Assad O, Hermann R, Lilla D, Mellies B, Meyer R, Shevach L (2011). Entertainment Computing—ICEC 2011.

[17] Dill S, Müller PN, Caserman P, Göbel S, Hoog Antink C, Tregel T (2024). Sensing in exergames for efficacy and motion quality: scoping review of recent publications. JMIR Serious Games.

[18] Li J, Theng YL, Foo S (2020). Effect of exergames on depression and anxiety in older adults: a systematic review and meta-analysis. J Med Internet Res.

[19] Chen MJ, Fan X, Moe ST (2002). Criterion-related validity of the Borg ratings of perceived exertion scale in healthy individuals: a meta-analysis. J Sports Sci.

[20] Johnson D, Gardner MJ, Perry R (2018). Validation of two game experience scales: the Player Experience of Need Satisfaction (PENS) and Game Experience Questionnaire (GEQ). Int J Hum Comput Stud.

[21] Ostrow KS, Heffernan NT Testing the validity and reliability of intrinsic motivation inventory subscales within assistments. https://link.springer.com/chapter/10.1007/978-3-319-93843-1_28.

[22] Armstrong RA, Eperjesi F, Gilmartin B (2002). The application of analysis of variance (ANOVA) to different experimental designs in optometry. Ophthalmic Physiol Opt.

[23] Wang C, Lee C, Shin H (2023). Digital therapeutics from bench to bedside. NPJ Digit Med.

[24] Montoya MF, Muñoz J, Henao OA (2021). Fatigue-aware videogame using biocybernetic adaptation: a pilot study for upper-limb rehabilitation with sEMG. Virtual Real.

[25] Li A, Qiang W, Li J, Geng Y, Qiang Y, Zhao J (2025). Retracted: evaluating the clinical efficacy of an exergame-based training program for enhancing physical and cognitive functions in older adults with mild cognitive impairment and dementia residing in rural long-term care facilities: randomized controlled trial. J Med Internet Res.

[26] Chen X, Wu L, Feng H (2023). Comparison of exergames versus conventional exercises on the health benefits of older adults: systematic review with meta-analysis of randomized controlled trials. JMIR Serious Games.

[27] Chen Y, Zhang Y, Guo Z, Bao D, Zhou J (2021). Comparison between the effects of exergame intervention and traditional physical training on improving balance and fall prevention in healthy older adults: a systematic review and meta-analysis. J Neuroeng Rehabil.

[28] Salti S, Schreer O, Di Stefano L Real-time 3D arm pose estimation from monocular video for enhanced HCI.

[29] Simonsen D, Popovic MB, Spaich EG, Andersen OK (2017). Design and test of a Microsoft Kinect-based system for delivering adaptive visual feedback to stroke patients during training of upper limb movement. Med Biol Eng Comput.

[30] Chen J, Yang T, He Q (2025). The impact of gamified interventions on the management of chronic obstructive pulmonary disease: systematic literature review. JMIR Serious Games.

[31] Chu CH, Biss RK, Cooper L, Quan AML, Matulis H (2021). Exergaming platform for older adults residing in long-term care homes: user-centered design, development, and usability study. JMIR Serious Games.

